# Strengths, Weaknesses, Opportunities, and Threats Analysis of the Use of Digital Health Technologies in Primary Health Care in the Sub-Saharan African Region: Qualitative Study

**DOI:** 10.2196/45224

**Published:** 2023-09-07

**Authors:** Niki O'Brien, Edmond Li, Cynthia N Chaibva, Raquel Gomez Bravo, Lana Kovacevic, Nana Kwame Ayisi-Boateng, Olivia Lounsbury, Ngnedjou Francoise F Nwabufo, Ephraim Kumi Senkyire, Alice Serafini, Eleleta Surafel Abay, Steven van de Vijver, Mercy Wanjala, Marie-Claire Wangari, Shabir Moosa, Ana Luisa Neves

**Affiliations:** 1 Institute of Global Health Innovation Imperial College London London United Kingdom; 2 Department of Nursing and Midwifery Sciences National University of Science and Technology Bulawayo Zimbabwe; 3 Africa Forum for Primary Health Care (AfroPHC) Bulawayo Zimbabwe; 4 Department of Cognitive and Behavioral Sciences University of Luxembourg Luxembourg Luxembourg; 5 Rehaklinik Centre Hospitalier Neuro-psychiatrie Luxembourg Luxembourg; 6 Department of Medicine Kwame Nkrumah University of Science and Technology Kumasi Ghana; 7 World Organisation of Family Doctors, Africa Region Kumasi Ghana; 8 Johns Hopkins Children’s Center Johns Hopkins Hospital Baltimore, MD United States; 9 Africa Forum for Primary Health Care Yaounde Cameroon; 10 Department of Health Sciences Adventist University Cosendai Yaounde Cameroon; 11 Family Health and Development Foundation Yaounde Cameroon; 12 One Health Network Yaounde Cameroon; 13 Africa Forum for Primary Health Care Accra Ghana; 14 GA West Municipal Hospital Accra Ghana; 15 Liverpool School of Tropical Medicine Liverpool United Kingdom; 16 University of British Colombia Okanagan, BC Canada; 17 Local Health Authority of Modena Modena Italy; 18 University of Modena and Reggio Emilia Modena Italy; 19 Department of Public Health University of Edinburgh Edinburgh United Kingdom; 20 Africa Forum for Primary Health Care Addis Ababa Ethiopia; 21 Department Family Medicine OLVG Hospital Amsterdam Netherlands; 22 Amsterdam Health & Technology Institute Amsterdam Netherlands; 23 Africa Forum for Primary Health Care Nairobi Kenya; 24 Department of Family Medicine University of Witwatersrand Johannesburg South Africa; 25 Johannesburg Health District Johannesburg South Africa; 26 Department of Primary Care and Public Health Imperial College London London United Kingdom

**Keywords:** digital health, digital health technology, telemedicine, remote care, primary care, primary health carel PHC, COVID-19, global health, sub-Saharan Africa, eHealth

## Abstract

**Background:**

Digital health technologies (DHTs) have become increasingly commonplace as a means of delivering primary care. While DHTs have been postulated to reduce inequalities, increase access, and strengthen health systems, how the implementation of DHTs has been realized in the sub-Saharan Africa (SSA) health care environment remains inadequately explored.

**Objective:**

This study aims to capture the multidisciplinary experiences of primary care professionals using DHTs to explore the strengths and weaknesses, as well as opportunities and threats, regarding the implementation and use of DHTs in SSA primary care settings.

**Methods:**

A combination of qualitative approaches was adopted (ie, focus groups and semistructured interviews). Participants were recruited through the African Forum for Primary Care and researchers’ contact networks using convenience sampling and included if having experience with digital technologies in primary health care in SSA. Focus and interviews were conducted, respectively, in November 2021 and January-March 2022. Topic guides were used to cover relevant topics in the interviews, using the strengths, weaknesses, opportunities, and threats framework. Transcripts were compiled verbatim and systematically reviewed by 2 independent reviewers using framework analysis to identify emerging themes. The COREQ (Consolidated Criteria for Reporting Qualitative Research) checklist was used to ensure the study met the recommended standards of qualitative data reporting.

**Results:**

A total of 33 participants participated in the study (n=13 and n=23 in the interviews and in focus groups, respectively; n=3 participants participated in both). The strengths of using DHTs ranged from improving access to care, supporting the continuity of care, and increasing care satisfaction and trust to greater collaboration, enabling safer decision-making, and hastening progress toward universal health coverage. Weaknesses included poor digital literacy, health inequalities, lack of human resources, inadequate training, lack of basic infrastructure and equipment, and poor coordination when implementing DHTs. DHTs were perceived as an opportunity to improve patient digital literacy, increase equity, promote more patient-centric design in upcoming DHTs, streamline expenditure, and provide a means to learn international best practices. Threats identified include the lack of buy-in from both patients and providers, insufficient human resources and local capacity, inadequate governmental support, overly restrictive regulations, and a lack of focus on cybersecurity and data protection.

**Conclusions:**

The research highlights the complex challenges of implementing DHTs in the SSA context as a fast-moving health delivery modality, as well as the need for multistakeholder involvement. Future research should explore the nuances of these findings across different technologies and settings in the SSA region and implications on health and health care equity, capitalizing on mixed-methods research, including the use of real-world quantitative data to understand patient health needs. The promise of digital health will only be realized when informed by studies that incorporate patient perspective at every stage of the research cycle.

## Introduction

Digital health technologies (DHTs) are defined by the World Health Organization (WHO) as a broad term that encompasses eHealth and other developing areas, such as advanced computer sciences use (ie, artificial intelligence, big data analytics, internet of things) [1]. DHTs have become essential resources in the health care system, transforming the delivery of services and products, with the aim to improve the health and well-being of the population [2]. The rapid growth of technologies and their integration have been exponential in the last decades [3], and the COVID-19 pandemic has accelerated its rise in many parts of the world and transformed it into a critical tool to monitor and fight the pandemic and provide care (eg, teleconsultation, e-prescription, and eHealth apps) [4].

DHTs are a promising solution to reduce inequalities, increase the accessibility of care, empower people, integrate different levels of care, and allow more effective health management, among others [5,6]. They play an important role in strengthening health systems and public health, working toward universal health coverage [2]. In addition, they play a crucial role in the provision of primary health care through teleconsultations among other initiatives. The WHO Africa Regional Office emphasizes the crucial role of people-centered health systems enabled by digital health solutions to be attained by 2026 [7].

DHTs can enhance health care delivery in several aspects, including improving access to care, especially for those in hard-to-reach areas; improvements in safety and quality of health care services and products, improving knowledge and access of health workers and communities to health information; improving efficiency and cost savings in health services delivery; providing a solution for the increasing shortage of health care staff; and improving social, economic, and environmental determinants of health, all of which could contribute to the attainment of universal health coverage [8,9].

Many of these cutting-edge technologies have not reached the areas that would profit most, especially low-income countries. This is the case in many regions in sub-Saharan Africa (SSA), where people at higher risk of many health conditions have the lowest access to health innovations [10]. The population is expected to double by 2050. This, together with the lower life expectancy and higher maternal mortality compared with the rest of the world, represents a huge challenge. According to previous literature, in the SSA region, digital health deployment has been constrained by some challenges, including suboptimal coordination of pilot projects [11], lack of awareness and knowledge about digital health, and poor infrastructures (eg, unstable power supply, poor internet connectivity, limited distribution of digital devices, and lack of interoperability of the numerous digital health systems) [8,11]. The literature is mostly concentrated around piloting DHTs [8,9].

This study aimed to expand on previous research on the subject and to specifically evaluate the current strengths and weaknesses, as well as the main opportunities and threats to implementing DHTs in primary health care in SSA, as seen by a wide cross-section of primary health care providers themselves and experts working across the continent.

## Methods

### Overview of the Methods Used

To meet the study’s objectives, a combination of qualitative approaches was used (ie, web-based focus groups and semistructured interviews). Focus groups are a qualitative method designed to capture both individual perspectives and group dynamics [12,13], and there is an established methodology for conducting them via videoconference [14]. In-depth interviews allow the interviewer to explore and examine individual perceptions in deeper detail and are therefore an effective method for interpretative inquiry [15]. A multidisciplinary team including medical doctors (ALN, EL, RGB, EKS, AS, MW, MCW, SM, and SV), nurses (CC), public health specialists (FN), and health services researchers (NO, OL, and LK) with previous experience on qualitative research performed this study. The COREQ (Consolidated Criteria for Reporting Qualitative Research) [16] checklist was used to ensure that the study meets the recommended standards of qualitative data reporting.

### Recruitment

Members of the African Forum for Primary Care (AfroPHC) [17] were invited to participate in the focus groups via email. The AfroPHC is a network of professionals working in primary care in the African region, with a membership of 635 people, including 40 out of the 53 countries in Africa. The members include a variety of professions and backgrounds, including nurses, doctors, community health workers, pharmacists, managers, and allied health professionals, among others. Participants for the interviews were recruited through the Institute of Global Health Innovation and AfroPHC networks and were invited to participate via email. In both cases, convenience sampling was used, and participants from mixed roles were included. Inclusion criteria were primary care professionals (clinical and nonclinical) with experience using digital technologies in primary health care in SSA. Participants were excluded if they did not have any involvement in primary care practice.

### Data Collection

Focus groups were conducted in November 2021, in partnership with AfroPHC and the World Organization of Family Doctors Working party on eHealth. Focus groups were conducted via Zoom (Zoom Technologies) and were 1 hour in length. Each focus group was led by 1-2 moderators who followed the topic guide developed in advance of the sessions (see Multimedia Appendix 1). All participants were invited to contribute to the session and were prompted to share their views if initially reluctant. Web-based interviews were conducted via Microsoft Teams (Microsoft Corp) between January and March 2022. Topic guides, including open-ended questions, were used to cover relevant topics in the interviews and focus groups, using a strengths, weaknesses, opportunities, and threats (SWOT) analysis approach [18]. The fill topic guides are presented in Multimedia Appendix 1.

SWOT analysis is a strategic planning technique used to help organizations to identify the main SWOT in a given context and is intended to identify the internal and external factors that are favorable and unfavorable to embracing the full potential of a solution or project, with the following definitions of each area [18,19]: strengths are the characteristics of the business or project that give it an advantage over others; weaknesses are the characteristics that place the business or project at a disadvantage relative to others; opportunities are the elements in the environment that the business or project could exploit to its advantage; threats are the elements in the environment that could cause trouble for the business or project.

The approach has been used widely in health research to assess the aspects of health care policy and provision to inform future planning [20-22]. While the SWOT analysis approach is better known for its use in organizations, the approach has also been used to analyze systems, specifically health systems [21,23]. In this paper, the SWOT analysis focused on the SWOT of using DHTs in health systems within the SSA region. The authors selected SWOT as the framework that enables participants in the research to share their views and experiences in a structured and intuitive way that can be replicated across separate interviews and focus groups. SWOT analysis is also a useful tool to capture relationships between internal and external factors in the present time and into the future, which is valuable in drawing conclusions in systems-level research.

Web-based interviews and focus groups were recorded and transcripts compiled verbatim. The transcripts were hosted on secure servers. All interviews and focus groups were conducted in English, and the interviewers had no previously established relationship with the participants of the study. Participants had minimal knowledge of the research team to minimize the potential for bias and assumptions.

### Data Analysis

Interview and focus group transcripts were systematically reviewed by a pair of 2 independent researchers using framework analysis. The framework analysis method has 5 main stages: familiarization, identification of a thematic framework, indexing, charting, and mapping and interpretation [24]. At every stage of the data analysis, the coding process was kept deductive and inductive, allowing the ongoing inclusion of any emergent themes that were not initially identified captured. All themes identified were supported by participants’ quotations. As the framework analysis progressed, no new themes emerged, suggesting that data saturation had been reached. Participants did not provide consent for further contact, and therefore it was not possible to ask them for feedback on the findings.

### Ethics Approval

Ethical approval was granted by Imperial College London’s Ethics Research Committee (21IC7269).

## Results

### Participant Characteristics

A total of 33 participants participated in the study (n=13 and n=23 in the interviews and in focus groups, respectively; n=3 participants participated in both). Participants represented a variety of roles, including physicians (n=9, 27%), nonclinical professionals in academia (n=4, 12%), nonclinical professionals in industry (n=4, 12%), nonclinical professionals in international nongovernmental organizations (n=4, 12%), nurses or nurse clinicians (n=2, 6%), nonclinical professionals in government (n=1, 3%), and other clinical professionals (n=1, 3%). As focus group participants self-categorized through a survey conducted on Zoom, those who did not complete the survey (n=8, 24%) were categorized as “unknown.” Participants covered all African regions (a full description is provided in Table 1). The 13 individuals who participated in the interviews represented a variety of countries, including countries across the African region (the Democratic Republic of Congo, n=1; Kenya, n=3; Nigeria, n=3; South Africa, n=2, United States, n=1, Ethiopia, n=1, Rwanda, n=1; and Uganda, n=1). The 23 individuals that participated in the 6 focus groups represented 13 countries in total (South Africa, n=5; Cameroon, n=3; Kenya, n=2; Nigeria, n=2; Sierra Leone, n=2; United States, n=2; Democratic Republic of Congo, n=1; Ghana, n=1, Greece, n=1; Mozambique, n=1; Republic of Congo, n=1; United Kingdom, n=1; and Zimbabwe, n=1). Participants from countries outside of the SSA either worked for international organizations with a presence on the continent or were individuals from SSA who lived abroad.

The SWOT analysis is presented across five overarching aspects: (1) patients, (2) providers, (3) health care systems, (4) legislation and regulation, and (5) technology and infrastructure (Table 2). A full list of participant quotations is presented in Multimedia Appendix 2.

**Table 1:** Description of interview and focus group participants (n=33) by professional role and region.

			Values
**Role, n (%)**			
	Physician	9 (27)	
	Nonclinical professional (academia)	4 (12)	
	Nonclinical professional (industry)	4 (12)	
	Nonclinical professional (international nongovernmental organization)	4 (12)	
	Nurse or nurse clinician	2 (6)	
	Nonclinical professional (government)	1 (3)	
	Other clinical professionals	1 (3)	
**Region (as categorized by the United Nations), n (%)**			
	Central Africa	6 (18)	
	Eastern Africa	8 (24)	
	Southern Africa	7 (21)	
	Western Africa	8 (24)	
	Other	4 (12)	

**Table 2:** Strengths, weaknesses, opportunities, and threats analysis.

Aspects	Strengths	Weaknesses	Opportunities	Threats
Patients	Accessibility and continuity of care Use and treatment adherence Affordability Patient satisfaction and trust	Poor digital literacy Health inequalities and digital divide	Improving literacy Improve patient empowerment and self-care Improve equity Tailored solutions and user-centered design	Patient buy-in
Providers	Collaboration Continuous professional development Decision-making	Lack of human resources Poor provider buy-in Lack of support and training High turnover rates	Enhancing access to secondary care advice Clinical support tools	Provider buy-in Lack of resources and local capacity
Health systems	Improved quality of care Data analytics and learning health systems Universal health coverage	High costs and inadequate funding Lack of coordination and fragmentation of services	Improve quality of care Knowledge generation (research, planning, and delivery) Optimization of resource and finances Learning from best practice and scaling up	Resistance to change Lack of governmental support Fraud and misuse Sustainability
Legislation and regulation	Governmental support and legislation Digital health regulation and frameworks	Lack or inadequate regulation	Improve governmental support Legal implications Data ownership and monetization	Restrictive regulations
Technology and infrastructure	Adoption and innovation fast-track	Lack of basic facilities and equipment Poor internet access Poor integration and interoperability	Improving basic infrastructure Data linkage	Cybersecurity and data privacy

### Strengths

**Figure 1:**
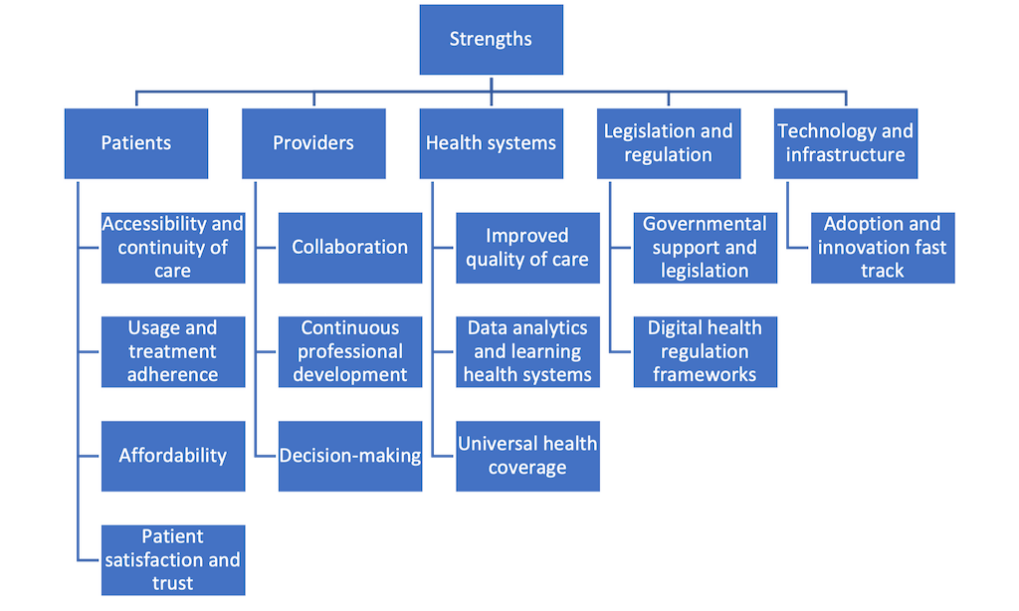
Concept map of strengths identified.

At the patient level, the role of DHTs in improving the accessibility of care emerged as a major theme (Figure 1). This was especially relevant in the case of hard-to-reach populations and during the COVID-19 pandemic as a means of ensuring both accessibility and continuity of care.

Technology...allows us to reach hard-to-reach places, like geographic and even demographic [segments] that otherwise we wouldn't be able to reach with traditional devices and methods.

Interview participant #6

It was also noted that improved accessibility increases usage and adherence. Affordability was also identified as one of the main strengths of DHTs in the African region, particularly in what concerns cost savings and financial efficiency for both patients and insurance schemes.

Participants also noted the role of DHTs in improving patient satisfaction and trust. They highlighted the convenience of remote care when addressing sensitive medical conditions (eg, sexual or mental health issues), and the role of integrated electronic health records and alerts in improving the patient’s trust (eg, by improving transparency and safety).

For providers, DHTs improved collaboration (both within primary care teams and within secondary care) and facilitated training and professional development, as well as improved treatment outcomes.

I’ve been in a situation where we’ve been able to reach out to a first-class paediatric cardiologist and he was able to give his opinion within a few minutes. And that changed everything for the baby.

Focus group participant #22

The ability to build the capacity and skill sets of health care providers, especially in rural, hard-to-access areas of Africa, was also reported to improve provider satisfaction and motivation. Another essential aspect identified was the role of DHTs on decision-making, particularly in what concerns the incorporation of digital support tools, standard operating procedures, and risk stratification tools.

From a health system perspective, DHTs were reported to enhance the quality of care across several domains. The use of automated reminders, integrated electronic medical records, and remote triage systems to support prioritization of patients were reported to improve care efficiency. Improved timeliness of care—made possible by allowing patients to easily access care without having to physically travel in-person for menial queries—as well as better coordination of care, and streamlined logistics were other strengths noted.

With DHTs, we know that we are having a stock out of supply in this area...it helps to provide services more rapidly and our response time is faster than if we were using other means.

Interview participant #2

Participants highlighted that DHTs also improve safety; examples included the use of apps to identify medication adverse effects and interactions and the use of task management to ensure patients and households in need are followed up. Another important aspect of quality improvement highlighted concerns about equity. Participants reported DHTs’ ability to allow patients to access health care despite their geographic location (eg, rural areas), with potential benefits on reducing existing health inequities and improving the quality of the care provided.

Identified strengths from a health system perspective also included the positive impact on building and supporting a learning health system culture, particularly using dashboards and monitoring platforms to inform continuous improvement. Finally, DHTs were identified as a critical aspect to achieving universal health coverage in the African region.

In what concerns legislation and regulation, participants noted positive developments in governmental support, policies, guidelines, and frameworks. The improvements in technology and financial infrastructure were also noted as strengths, underscored by reliability, availability, relative affordability, and penetration of use in the African region. Innovation being fast-tracked was noted across several technologies including telephone, SMS services, mobile apps, and financial services including digital payment platforms.

I know that in my own country, Nigeria, I will say practically everybody has a mobile phone...So that’s it, it is a huge opportunity.

Focus group participant #22

### Weaknesses

**Figure 2:**
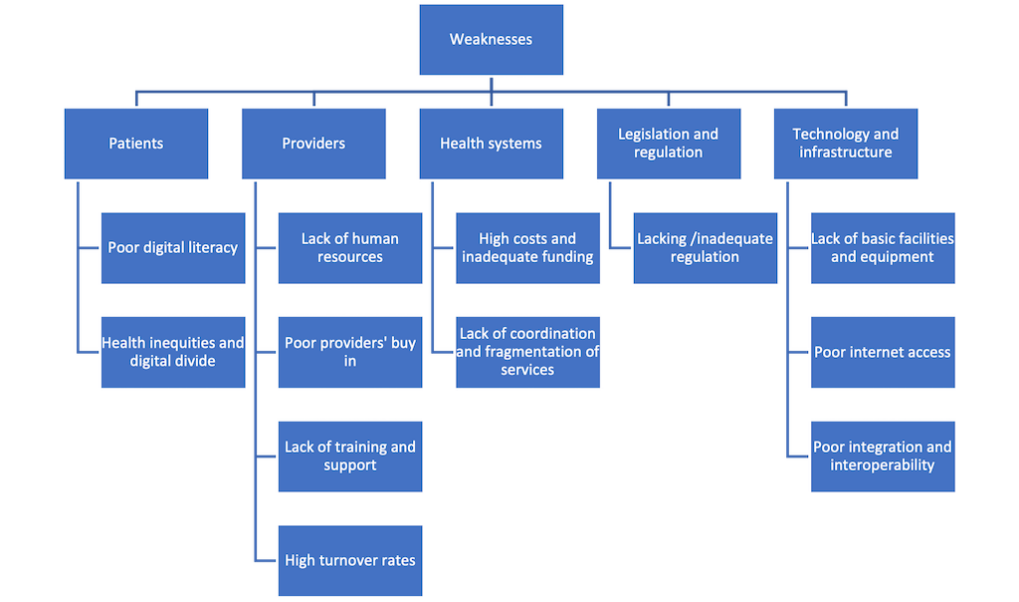
Concept map of weaknesses identified.

At the patient level, the main weaknesses reported were poor digital literacy and the potential of DHTs to entrench existing inequities and exacerbate the digital divide (Figure 2).

A larger percentage of people who live in those areas are illiterate. They haven’t gone to school, and they’re not fluent with mobile technology, or technology in general.

Focus group participant #16

Health inequities manifesting in the form of digital and health illiteracy often mirror the wealth divide between many rural and urban African settings. Participants remarked that the affluent, city-dwelling populations (with higher levels of education, health awareness, and digital literacy) are best placed to take advantage of the growing number of DHTs being introduced. On the other hand, poorer, rural communities—which stand the most to gain from remote care tools—often remain unable to maximize the use. Participants also noted the challenges associated with follow-up after digital-enhanced care. They stressed that even if a patient can access and use a remote consultation, required follow-up (ie, diagnostic test or medical intervention) would often necessitate having a face-to-face appointment. Participants noted that this blunted the perceived value and overall benefit of the technology for some patients.

From the health care provider’s perspective, the lack of human resources, poor buy-in, and lack of training and support were prominent weaknesses surrounding the use of digital technologies in the African health care landscape.

Most of the workers working in primary health care...didn’t really have a tertiary education in health. They are quickly trained for just a day, or some are trained for six months, some are trained for a year...During their training digitisation is not really included.

Focus group participant #21

From a system perspective, participants highlighted weaknesses such as the high costs associated with equipment purchase, running, and maintenance. These costs go beyond upfront costs for the immediate health care facility as they often encompass upgrades to the underlying infrastructure (eg, reliable internet service) and training necessary for staff to effectively use DHTs. Despite the growing affordability of mobile phones in Africa, data connectivity often remains prohibitively expensive for most, with SMS services still being the most used. Participants described these issues alongside other aspects of funding, including insufficient allocation and inappropriate usage of the funds available. Participants also mention the lack of coordination between the range of stakeholders involved, often resulting in duplication of digital solutions and fragmentation.

There are many players.... The biggest challenge is it is a fragmented market, it’s a huge value-chain, and each part of the value chain is a specialised system, and all the systems talking together is in fact, a global problem that needs to be addressed.

Interview participant #8

Participants identified several weaknesses relating to inappropriate or absent regulations for the use of DHTs; they also noted that regulations for use are often not aligned with national-level guidance, creating discrepancies and uncertainty for providers.

It’s the life of people so we cannot wait until we see an accident occurring. [We need] strong regulations about DHTs, what are the limits, what are the roles and regulations and responsibilities of each party, and who is going to enforce it to ensure that when things don’t go well, who is going to fix it and pay for it.

Interview participant #7

Finally, in what concerns weaknesses pertaining to technology and infrastructure, participants highlighted that many African contexts have more pressing issues that need to be solved before advancing digital technologies. They noted the lack of basic facilities (eg, water and power) and equipment (eg, modern computers and mobile phones, and reliable high-speed internet). Another important theme identified was the poor integration of existing systems and poor interoperability between already available solutions.

### Opportunities

**Figure 3:**
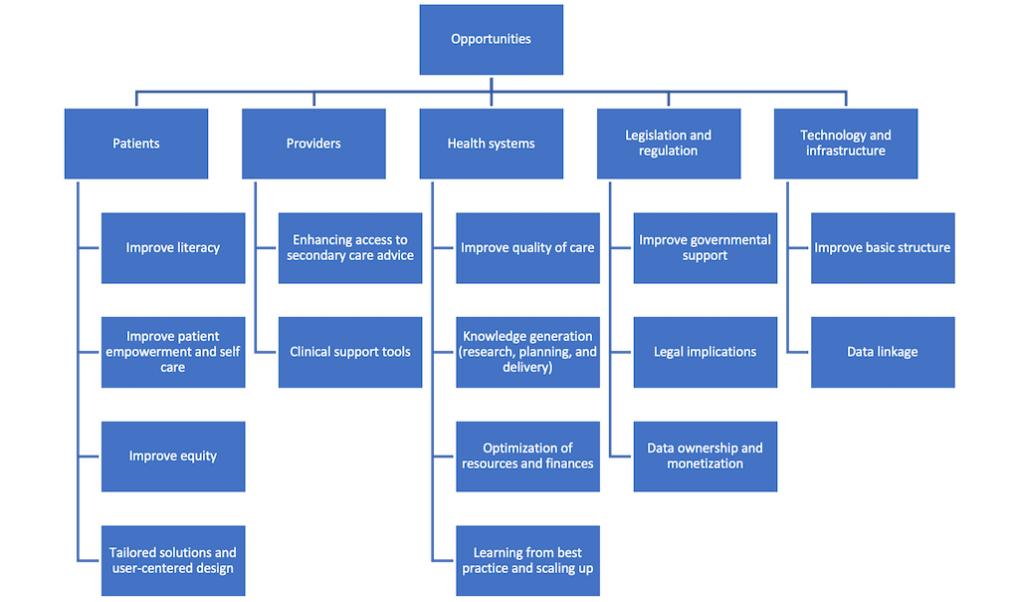
Concept map of opportunities identified.

Participants noted emerging opportunities to improve patients’ digital and general literacy (Figure 3). Other relevant opportunities mentioned were the role to be played by DHTs in enhancing patient empowerment and self-care through the use of wearables and remote monitoring tools (ie, pulse oximetry, blood pressure monitors, glucometers, thermometers, and digital stethoscopes).

It was also noted that moving forward, there is a need to develop tailored digital interventions that answer to the needs of specific patient groups, capitalizing on user-centered design approaches. Finally, the underpinning concern for patients is the need to embed strategies that improve equity and reduce—rather than entrench—existing health disparities.

For providers, the main opportunities identified included enhancing access to secondary care advice and guidance and the use of DHTs as decision support tools. The role of artificial intelligence to support clinical decision-making, standardize care by best practice, and automate checks were some of the examples covered by participants.

It will also...improve quality because you can be able to get a second opinion from specialist doctors using digital health, to be able to support the care in patients so that the quality is almost the same in urban and rural areas.

Focus group participant #17

Second, participants consider that there is a future opportunity for digital technologies to support the optimization of resources and finances, including more responsible use of existing resources, reduce health care costs, and increase financial transparency. Pragmatically, this was envisaged through the role of digital innovation supporting preventative care and encouraging greater economic empowerment of more segments of the population over time. Participants noted that if implemented and used appropriately, digital health tools could help transform large parts of Africa from their dependency on aid to a self-sustainable health care ecosystem.

For health systems, quality improvement in the context of direct patient care was highlighted as one of the main future opportunities. However, the opportunity is wider: secondary uses of health care data and data analytic approaches can generate new scientific knowledge and inform service delivery and planning. Participants also noted an important opportunity in the ability to learn from best practice in digital innovation, including the possibility for shared learning from international experiences. There is an opportunity for DHTs to optimize financial resources, both by automating manual processes and by supporting preventative care (ie, reminders and self-management).

For me, one point is that [DHTs] have the potential to shift, especially in Africa context,...healthcare or the health systems, from the perspective of an aid corridor into a source of economic empowerment.

Focus group participant #15

From a regulatory perspective, there are opportunities to improve governmental support and regulations, particularly in the context of liability when using DHTs.

For example, in telemedicine....When do you refer the patient? When do you prescribe on [the] phone? What if anything goes wrong? You will be liable as a clinician. So, government will need to regulate such practice, so that we know where to draw the line.

Interview participant #11

Interestingly, participants also mentioned opportunities around data ownership and monetization (ie, trading own health information) that could be made clearer through regulation.

In line with the weaknesses previously identified, notable opportunities also include greater government investment in improving the basic infrastructure and creation of unique patient identifiers to facilitate data linkage, and ultimately improved interoperability.

A unique identifier in your civil registration number identifier should make its way into every piece, from pathology specimen request to every clinical record.

Interview participant #10

### Threats

**Figure 4:**
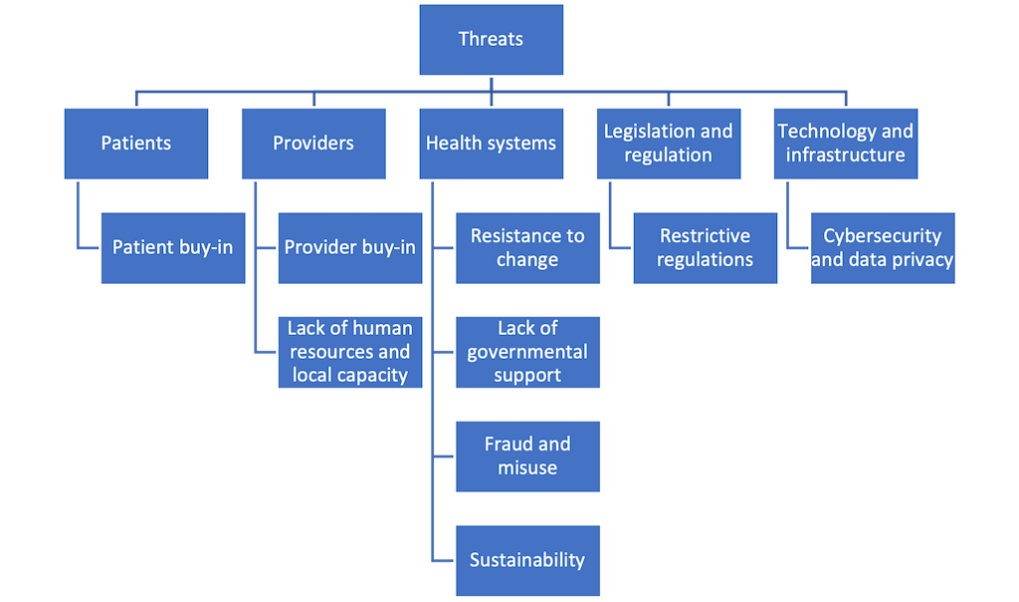
Concept map of threats identified.

Poor stakeholder buy-in (both from patients and providers) was noted as a major threat to the use of DHTs in Africa (Figure 4). Lack of human resources and local capacity were previously described as current weaknesses, and current projections suggest that they will continue to represent a growing threat in the future. Proposed strategies to overcome stakeholder buy-in included producing robust evidence, education, and advocacy.

Resistance to change, as well as lack of governmental support, was also noted as important future threats. Lack of governmental support was often described in association with the lack of a strategic vision, lack of political willingness to support implementation, and lack of investment to address the aforementioned basic infrastructure shortcomings.

One of the barriers would be political will. A lot of people talk, and they don’t walk their talk - this is amazing, but they don’t truly prioritise it.

Interview participant #9

Participants also noted that fraud and misuse, particularly in the context of potential gaming behaviors, represented a substantial threat. For example, participants felt it is simpler for individuals to impersonate other people, in order to gain access to health services or prescriptions, via DHTs. Sustainability challenges were linked to high turnover rates of trained staff and presented as a significant limiting factor to sustainable capacity building. There were also important concerns about the long-term sustainability of digital initiatives, as digital interventions are often confined to the life cycle of donors.

We have funding to implement electronic medical records (EMR) for five years, and then the project’s over. We don’t know if they’re actually still using it, or what their [situation is] like. You’re at risk of all that investment and those five years of trying to create an EMR, just to kind of going down the drain.

Interview participant #1

The absence or inadequacy of regulations was previously noted as a weakness; participants also consider it will represent an important threat in the future, particularly if these regulations are restrictive and hinder innovation.

And at some point, the regulation will be created and it will probably be far more constricted, that’s what we’re expecting at least, than what systems have been doing, and we will all have to pull back to meet the regulations, probably to the disadvantage of the patient at the end of it.

Interview participant #3

Due to a lack of transparency, the lack of standardization of equipment, patchwork of systems and infrastructure used, as well as the often ad hoc nature of their implementation, concerns regarding data privacy and security were commonplace among both health care providers and patients.

For example, when something happens or when a disease breaks out in a certain area, and we are doing surveillance or something, communities mostly ask you: “Where are you taking our stories? Where are you taking our information? There’s a bit of a concern.”

Focus group participant #16

## Discussion

### Key Findings

The strengths of current DHTs described by participants included improved accessibility and continuity of care, usage and treatment adherence, affordability, and patient care satisfaction and trust. For providers, DHTs allowed for greater collaboration, continuous professional development, and supported decision-making. From a health system perspective, using DHTs improved care quality, supported learning health systems, and contributed to further universal health coverage. From the legislation and regulation perspective, participants reported the governmental support and legislation and regulatory frameworks as strengths of current DHTs use. From a technological and infrastructure perspective, participants reported that widespread adoption and innovation fast-track were significant strengths of DHTs in the SSA setting.

Regarding weaknesses for patients, the use of DHTs was often constrained by poor digital literacy and health inequalities. The lack of adequate human resources, provider buy-in, sufficient support and training, and high staff turnover rates were also noted. At the systems level, the overall high cost of DHTs and the lack of coordination in systems implementation and resource allocation were also identified as weaknesses. Other aspects identified included the lack of or inadequacy of existing regulations, lack of basic facilities and equipment, unreliable internet access, and poor systems integration and interoperability.

Future opportunities include improving patients’ digital literacy, empowering patients for self-care, increasing equity, and ushering in greater patient-centric design to develop tailored solutions. For health care providers, there is an opportunity for DHTs to enhance access to secondary care advice and provide clinical support. For systems, DHTs can represent an opportunity to improve the quality of care, generate new clinical knowledge, and optimize health care planning and expenditures. Learning from international best practices was an important opportunity noted. There are also opportunities to improve government support, legal implications, data ownership and monetization, improving the underlying digital infrastructure, and encouraging data linkage.

Major threats identified include suboptimal patient and provider buy-in, lack of human resources and local capacity, resistance to change, lack of governmental support, fraud and misuse, sustainability, restrictive regulations, and threats to cybersecurity and data privacy.

### Comparison With Previous Literature

The themes highlighted by our study were largely congruent or complemented many of the findings from other contemporary studies exploring the use of DHTs in the SSA region.

DHTs, including telemedicine, were used widely in primary health care during the COVID-19 pandemic to facilitate health service delivery and contributed to improved health outcomes [25,26]. Concerning the strengths of DHTs and virtual care usage, Mbunge et al [27] performed a systematic review investigating the experiences in South Africa during the COVID-19 pandemic. The extensive use of mobile phone–based solutions, particularly SMS, was instrumental in ensuring the continuation of routine and pandemic-related health care services. Reported strengths from DHTs ranged from the ability for patients to conveniently renew prescriptions remotely, reduced prescription errors, and improved overall medication safety to facilitating patient counseling and arranging medication deliveries to ensure medication adherence.

Our study’s findings were also consistent with the array of weaknesses limiting DHTs in SSA settings at the patient and provider levels previously reported in the wider literature [28,29]. Mbunge et al [27] outlined problems including the tendency for patients to miss appointments, the inability for certain services such as mental health care to be delivered, and language barriers. Similarly, Oseni et al [26] noted that in the Nigerian context during the COVID-19 pandemic, around 60% of patients could use the telephone as a means of contacting their family physician, indicating similar health care access and equity challenges identified through our research. Problems concerning the lack of follow-up care, similar to what was identified in our study, were highlighted by Mbunge et al [27], as a substantial barrier to preventing some patients from being able to independently manage their own health conditions without resorting to in-person consultations [27].

A recent study by Motiwala and Ezezika [30] described system-level weaknesses concerning the scaling up of the use of DHTs in Ethiopia, Nigeria, and Rwanda. These spanned from the insufficient number of health care providers trained on the use of DHTs to inadequate availability and accessibility of basic health care equipment in underfunded facilities. “Brain drain” as a result of low remuneration was reported to be a notable contributor to ineffective retainment of skilled staff and providers tended to emigrate to other countries where health care equipment, including DHT, was readily available, they were better paid, and felt their work was more valued. Other related weaknesses at the systems level mentioned in the literature included a general lack of understanding and ineffectual policies supporting the use and expansion of DHTs and a volatile political climate, as well as high costs of digital infrastructure [30,31]. Often, these were further compounded by the poor underlying basic infrastructure (eg, unreliable electricity and clean water supplies, and low digital network access and coverage).

Despite challenges, data linkage and clinical support tools, both identified as opportunities, are increasingly being used to develop and validate machine learning technology to support health care workers and improve patient care across SSA. For example, THINKMD’s mobile health clinical risk assessment platform has been tested and validated using clinical data and malaria risk assessments acquired from children presenting to health clinics in Kano, Nigeria [32]. The machine learning algorithms identify malaria cases using symptom and location data, paving the way for implementation for such technology to be used to identify children with malaria or nonmalaria attributable febrile illnesses. As cybersecurity and data privacy was identified as a threat to advancing DHT, it will be important for new technologies to demonstrate their security and adherence to international standards in order to build public trust and buy-in.

The opportunities identified through our research align with global efforts to support DHTs in SSA [33]. Learning from best practice to aid DHT scale up was identified as a theme and is echoed in current and planned work at national and international levels. For example, the WHO Digital Health Platform [34] is an open-source public good providing digital solutions for electronic management of patient health records, supported by information and communications technology infrastructure principles, and includes a framework for sustainable implementation and capacity building [35]. The Digital Health Platform will evolve and mature over time depending on the country’s needs, context, and complexity. Additionally, existing international collaborations have recently been leveraged to develop practical guidance for economic evaluation of DHTs highly relevant to SSA, including the World Bank Framework for the Economic Evaluation of Digital Health Interventions [36]. Such a focus on economic impact will have positive implications for the optimization of resources and finances while potentially addressing the threat of sustainability identified.

As much of the existing literature was conducted prior to 2020, the findings of our study highlight that use of DHTs in SSA during the COVID-19 pandemic continued in line with prepandemic progress. This unique finding helps highlight which areas of use in SSA need to be prioritized and provides a clearer understanding of the challenges faced during the pandemic period.

### Strengths and Limitations

Our study provides a comprehensive evaluation of the strengths, weaknesses, opportunities, and threats related to the use of DHTs across African health systems. This is of particular interest due to their wider implementation following the COVID-19 pandemic, with timely findings enabling said health systems to make the most of future opportunities in this space. Drawing on the perspectives from a diverse group of participants is a key strength of this study. The participants represent a variety of health care roles and 16 countries, with all participants having direct experience of SSA health care settings. This ensured the identified themes and perspectives represent a wide range of experiences—both geographically and professionally. Furthermore, focus group facilitation and data analysis were performed by a multidisciplinary qualitative research team, including medical, public health, and health services researchers, ensuring expertise across the disciplines relevant to the topic of research.

Some limitations must also be noted. First, we considered a broad spectrum of DHTs which are heterogeneous in nature, requiring a careful interpretation of the findings and resulting in a potential lack of transferability to other settings. This limitation is difficult to avoid, given the number and variety of DHTs in use across global health systems, especially in the context of their rapid rollout during the COVID-19 pandemic. As a result, the structural features and relative level of development of the health system to which the findings are being applied to must be carefully considered. Another limitation stems from the self-selection of study participants, which has further implications for the interpretation of findings. Participants with more experience and knowledge of DHTs, or those holding a more positive view of their future potential, may have been more likely to opt into the study, therefore biasing the findings. Similarly, web-based focus groups and interviews may have excluded potential participants who were unable to access the web-based video conferencing platforms due to limited network connectivity, electricity, and so forth.

### Implications for Research, Practice, and Policy

Future research should explore the nuances of these findings across different technologies and settings across the SSA region. Patient perspective is a key aspect that must be embedded in any attempt to implement DHTs; therefore, future research should additionally focus on capturing the experiences of patients and caregivers. Subsequent research efforts should also use quantitative approaches, including Quantitative Content Analysis which can provide further insights into the themes highlighted in our study, as perceived by different stakeholders and in different contexts. This may equally include primary data collection methods using relevant performance indicators or analyzing secondary data sets of appropriate proxy measures and mapping the findings against standardized quality of care frameworks. Doing so would help better define the scope and nature of the inherent problems surrounding the use of DHTs indicated by our participants, allow for ongoing monitoring of quality-of-care improvements, and guide more effective allocation of resources for policy makers.

Previous research has highlighted the potential for DHTs to become a new determinant of health, with implications for equity [37]. As such, a key area in need of further investigation, as highlighted by our research, is the use of DHTs in urban versus rural populations in SSA and its implication on health and health care equity. As mentioned by many participants, the benefits and challenges associated with supporting DHTs and their usage by patients differ quite substantially between these settings. A more granular, quantitative understanding of patient health needs addressable by DHTs, such as care access patterns and what underlying factors are contributing to the challenges in using DHTs, is likely to be valuable to a more successful implementation and sustainable application. Only by doing so can DHTs be used to their fullest potential, bespoke to the unique context of SSA, and effective in tackling the needs of patients moving forward. Further investigation would enable greater insights into the use of DHTs across health systems (eg, community, primary, secondary, and tertiary care) to identify where DHTs are likely to be most valuable, particularly in relation to health care access and equity. In this regard, understanding more about DHTs and implications for patient experience and safety at transitions of care is highly important. Such investigation also offers the opportunity to consider the potential of priority setting and targeted investment as DHTs are scaled up toward the achievement of Universal Health Coverage by 2030 [38].

The research has highlighted the complex challenges of developing legislation, regulation, policy, and guidelines for DHTs as a fast-moving health delivery modality, which must be addressed to drive their use at scale. Effective regulation and policy are representative of government attention and buy-in to the topic, and as such, it is the responsibility of all stakeholders involved in the development and implementation of DHTs to advocate for the development of such guidance. In turn, it is the responsibility of national and local governments to engage in meaningful dialogue with stakeholders from across sectors to determine how regulation and policy can meet needs on the ground. The more fundamental roadblock is the human and financial resources required to keep pace; however, efficient and effective policy making for DHTs need not be seen as an unachievable task.

## Appendix

Multimedia Appendix 1 Topic guides.

Multimedia Appendix 2 Participant quotes.

## Abbreviations

AfroPHC: African Forum for Primary Care
COREQ: Criteria for Reporting Qualitative Research
DHT: digital health technology
NIHR: National Institute for Health and Care
SSA: sub-Saharan Africa
SWOT: strengths, weaknesses, opportunities, and threats
WHO: World Health Organization

## Data Availability

All data generated or analyzed during this study are included in this published article and Multimedia Appendix files.
