# Supplementary material for: Strengths, Weaknesses, Opportunities, and Threats Analysis of the Use of Digital Health Technologies in Primary Health Care in the Sub-Saharan African Region: Qualitative Study
Source: J Med Internet Res. 2023 Sep 7;25:e45224. doi: 10.2196/45224 (PMC10514769; doi:10.2196/45224)
Supplement: Multimedia Appendix 1 [file jmir_v25i1e45224_app1.docx]

Multimedia Appendix 1: Topic guides

***A. Topic Guide (Focus Groups)***

*Digital technologies*

Introduce the concept: We will use the following scope of “digital technologies” to frame the session:

“Digital technologies can include a range of solutions used to support the delivery of healthcare. These can include telephone, video, websites, chats applications, mobile apps, SMS messaging, chatbots, among others.”

*Topic guide questions*

What digital health technologies do you use in primary health care and how do you use these digital health technologies in primary health care?

Prompt – Brainstorm

What are the benefits of these digital health technologies in primary health care?

Prompt – What are the current strengths? What are the future opportunities? Are the benefits in clinical service or education?

What are the challenges in using digital health for primary health care?

Prompt – What are the current weaknesses? What are the major threats to reach its future potential? Are the challenges in capacity or systems?

***B. Topic Guide (Semi-structured interviews)***

*PART I: Individual experiences*

Can you tell me a bit about yourself?

Probe: work, expertise, areas of interest

2. Can you tell me about examples of using digital technologies in your work?

Probe: for a particular disease or in a particular treatment area?

Probe: what type of intervention (decision support, health screening, service delivery etc).

*PART II: SWOT*

Thinking about your work, but also the use of digital technologies used more widely in your country, how are digital technologies being used in primary health care?

Probe: EHR for direct patient care? Telehealth (monitoring, prevention, treatment)? Use of EHR for data mining (knowledge generation, public health, prediction, artificial intelligence)? decision-support tools? ePrescribing/eAppointments? mHealth (mobile health devices, apps)?

What are the benefits of these digital technologies in primary health care?

Probe: What are the current strengths? What are the future opportunities?

What are the challenges in using digital health for primary health care?

Probe: What are the current weaknesses? What are the major threats to reach its future potential?

Closing (1-2 minutes)

Anything else you would like to add?
