# Supplementary material for: Strengths, Weaknesses, Opportunities, and Threats Analysis of the Use of Digital Health Technologies in Primary Health Care in the Sub-Saharan African Region: Qualitative Study
Source: J Med Internet Res. 2023 Sep 7;25:e45224. doi: 10.2196/45224 (PMC10514769; doi:10.2196/45224)
Supplement: Multimedia Appendix 2 [file jmir_v25i1e45224_app2.docx]

Multimedia appendix 2: Participant quotes

***Thematic analysis of strengths identified***

*1. Patients*

*1.1. Accessibility & continuity of care*

*"Technology (...) allows us to reach hard to places, like geographically and even demographics that otherwise wouldn't be able to reach with traditional devices and methods” Interview participant 6*

*1.2. Utilisation and treatment adherence*

*"Someone who has easy access to healthcare on their phone they’re more bound to use it", Interview participant 9*

*1.3. Affordability*

*"It saves money for the people who were supposed to travel, the time, the cost of everything plus obviously the treatment itself. If you were to do all the cost savings, from the consumer perspective, it’s huge", Interview participant 7*

*"We have seen if you’re able to provide a digital product, it’s much cheaper for the insurance to distribute so they can give us prices as low as $40 per annum for a substantial cover that you can cover in an emergency", Interview participant 8*

*"And on the cost side because it’s just far cheaper, even if you have to buy a phone and a sim card and data, and call a number, it’s still cheaper than a consultation with a doctor”, Interview participant 3*

*1.4. Patient satisfaction and trust*

*“And we actually did a client satisfaction survey and actually they were very, very pleased compared to the traditional way”, Interview participant 7*

*"Young people who want to talk about potentially pregnancy or contraceptive use or whatever, they don’t even necessarily want to see the doctors face", Interview participant 3*

*2. Providers*

*2.1. Collaboration*

"*Some of our faculty may not be on the ground during the time of the meeting. Unlike in the past where that would have meant they will not participate in the meeting, now even those faculty members who are not on ground they can join virtually, so that has improved the practice”, Interview participant 11*

*“I've been in a situation where we've been able to reach out to a first-class paediatric cardiologist and he was able to give his opinion within a few minutes. And that changed everything for the baby”, Focus group participant 22*

*2.2. Continuous professional development*

*“For example, we have an application (…) [that allows] healthcare workers, especially community healthcare workers in rural areas, to access training through their mobile device. (…) You don't need to gather these people around and train them. They are given basic and essential education on so many different things in the community”, Focus group participant 16*

*“If we look at it from the staff perspective, we can use digital health to improve their capacity and their skills and knowledge - and that keeps them well motivated”, Focus group participant 22*

*2.3. Decision making*

*“It’s very useful to be able to have decision making tools, (…) and calculate very quickly the risk for a pulmonary embolism (…), or what is the mini-mental status of this patient”, Interview participant 13*

*“Through these digital technologies, we are able to visualise data from the various indicators, such that people at different level are able to use this for decision-making”, Interview participant 12*

*3. Health systems*

*3.1. Improved quality of care*

3.1.1. Efficiency

*"The application will automatically remind you of the tasks that you need to complete, those that are overdue, those that are coming up really soon, and then you're able to prioritise as a community health worker, and you plan your schedule for the week”, Interview participant 12*

*"People present very late for care generally, and they do that in large part because of the inconvenience of having to take time out of work, travel to a facility, wait for an appointment, see a doctor, get a prescription or a lab request, and then go to a lab, wait again, go to a pharmacy, wait again, be out of pocket (...) Telemedicine takes [that] away … it doesn’t take away the entire process, but it can address 70-80% of the inefficiencies in that system", Interview participant 3*

3.1.2. Timeliness

*"It reduces the waiting time, and then it makes the consultation faster.” Interview participant 11*

*" With DHTs, we know that we are having a stock out of supply in this area (…) It helps to provide services more rapidly and our response time is faster than if we were using other means.” Interview participant 2*

3.1.3. Safety

*“In terms of patient safety, it’s also very important (…) I [can] put up my drug app and I throw in all the drugs [the patient is taking], and it flags potential for adverse effects”, Interview participant 13*

*"These digital tools are helping VHTs (village health teams) to manage their tasks better because if you look at the registers, and if you are a VHT and you want to follow up, it means you have to flip through the pages, and there are chances of you missing out certain households”, Interview participant 12*

3.1.4. Equity

*“… beneficiaries [can] receive the same quality of care, no matter where they are located… [also,] you can be able to get a second opinion from specialist doctors using digital health”, Interview participant 3*

*"This is not something only for rich people or only for urban populations because they have clinics around. It’s reaching the poor and also that the poor are remote, in rural areas" Interview participant 7*

*3.2. Data analytics and learning health systems*

*“(…) dashboards help us monitoring performance, so we are able to know which community health workers are within our catchment area, and how they are performing. And then based on that performance, we can [provide] incentives”, Interview participant 12*

*"For me it has helped me design a learning system to track what exactly is happening in my clinic and over time say, am I improving or am I able to improve the quality of care that I’m offering my patients", Interview participant 13*

*3.3. Universal health coverage*

*"Digital health is plugging (…) or is a very important piece of a puzzle that’s been missing for a long time in our goal for primary healthcare and universal health coverage”, Interview participant 3*

*“(…) we are able to get the data from all over the country on time, and it will be easier to make accurate and fast decisions as to how universal coverage can be better managed”, Interview participant 3*

*4. Legislation and regulation*

*4.1. Governmental support and legislation*

*“These are actually government policies and guidelines. So, there’s the element of ownership”, Interview participant 2*

*"The government seems to want to have a long-term relationship. Now they want to expand the package of services they’re offering”, Interview participant 7*

*"One of the government policy strategies actually emphasises and encourages innovation and digital health is actually part of government”, Interview participant 2*

*4.2. Digital health regulation and frameworks*

*“The regulations are improving every day”, Interview participant 5*

*“Rwanda has (…) been working on this for many years so they have a very strong regulatory framework”, Interview participant 7*

*“Since COVID, we’ve seen a real shift with direct service delivery because it’s the first time it’s been regulated and permitted, at least in Kenya by the Ministry of Health, and by the Medical Board", Interview participant 3*

*5. Technology and infrastructure*

*5.1. Technology adoption and fast track*

"*Access to Internet connectivity is as high as 60% or 70% (…) Even pastoralists that are moving from place to place (…) have access to a phone", Interview participant 2*

*“From my experience, I've seen people using things as simple as WhatsApp, you know, to have a conversation”, Focus group participant 8*

*“I know that in my own country, Nigeria, I will say practically everybody has a mobile phone, practically anywhere you go people even have two or three answers, they call it answers, right? So that's it, it is a huge opportunity. But we have to be trite, if we really want to get it right”, Focus group participant 22*

***Thematic analysis of weaknesses identified***

*1. Patients*

*1.1. Poor digital literacy*

*“A larger percentage of people who live in those areas are illiterate. They haven't gone to school, and they're not fluent with mobile technology, or technology in general”, Focus group participant 16*

*1.2. Health inequities and digital divide*

*“Technology is present. (…) Whether there’s equality of access is a different issue”, Interview participant 13*

*“What I realise is mostly people within the urban areas are profiting from such services, and those are exactly the people that already have access to health service. (…) So now we have the educational biases, the academic literacy and technological literacy (...) Those are the issues that have to be ironed out”, Focus group participant 15*

*“[We] have to intentionally design digital health interventions for the rural community. When you design it from that perspective, it is easy to now get similar implementation within the rural community (…) the discrepancies are quite huge, (...) and the context has to shape the intervention we are trying to provide”, Focus group participant 15*

*2. Providers*

*2.1. Lack of human resources and capacity*

*“[We] don’t have the adequate number of staff. The few present are poorly motivated, and poorly trained, poorly supervised (…) Do you know that many of them don’t get paid regularly? (…) So how do you, we now start talking about bringing technology into that kind of environment? You cannot run Primary Health Care, a facility or any institution, if you don’t have the right people, and the right number, with the right mindset”, Focus group participant 24*

*2.2. Providers’ buy-in*

*“Doctors and nurses at the frontline of care don’t have much time to be punching computers because their duty is to serve and save lives”, Interview participant 4*

*“When there have been attempts to introduce electronic health records in similar settings that, I’ve had family physicians complaining that it prolongs their consultation time”, Interview participant 13*

*2.3. Lack of support and training*

*“First of all, people lack support, capacity building and training”, Focus group participant 16*

*“One challenge that we have is the inability of many personnel to use the software. (…) It always warrants training and supportive supervision, for the people at the operational level to get it right”, Focus group participant 20*

*“Most of the workers working in primary health care (…) didn't really have a tertiary education in health. They are quickly trained for just a day, or some are trained for six months, some are trained for a year [..] During their training digitisation is not really included”, Focus group participant 21*

*3. Health systems*

*3.1. High costs & inadequate funding*

*“[The] implementation costs [and] operational costs are quite challenging. Like I mentioned we need to depend on outside [funding] as well because some of these are very expensive.” Interview participant 2*

*3.2. Lack of coordination and fragmentation of services*

*“There are many players. (…) The biggest challenge is it is a fragmented market, it’s a huge value-chain, and each part of the value chain is a specialised system, and all the systems talking together is in fact, a global problem that needs to be addressed”, Interview participant 8*

*“We realise that personnel are always moving. People (…) are using [digital technologies] and then in a short while, probably they are transferred or (…) they need to move and then somebody else has to step in, then the challenge still surfaces”, Interview participant 4*

*4. Legislation and regulation*

*4.1. Lack / inadequate regulation*

*“Where do I store my data? Where do I do this and this? How do I get licensing? All those little touch points I think that it would be very great to clarity over regulation which allows companies to play”, Interview participant 9*

*We abide by regulation standards for use, but this doesn’t always (…) match the national regulations and standards. (…) So there absolutely should be more regulation”, Interview participant 1*

*It’s the life of people so we cannot wait until we see an accident occurring. [We need] strong regulations about DHTs, what are the limits, what are the roles and regulations and responsibilities of each party, and who is going to enforce it to ensure that when things don’t go well, who is going to fix it and pay for it”, Interview participant 7.*

*5. Technology and infrastructure*

*5.1. Lack of basic facilities and equipment*

*“Digital Health is good, [but] 70 to 80% of the Primary Health Care systems in African countries, including my own country, Nigeria, are dysfunctional. So, how can we build something like this (…), if some of the hospitals have no water, no running water, no power, no internet”, Focus group participant 22*

*“Another big challenge is the power supply. Power supply in Nigeria is erratic. So, most of the time we have electricity at this moment and the next moment it's off. So, what happens when there is no electricity supply for some time and then the system breaks down?”, Interview participant 11*

*“Community health workers work in environments where they do not even have access to power. They don't have where to charge these phones from, and will usually take their phones to public charging points, where they often get stolen”, Interview participant 12*

*5.2. Poor internet access*

*“[Often], the issue of having a mobile phone is not a problem. The main problem is getting through to them [patients], especially when network is poor”, Focus group participant 17*

*“Another problem is access to the internet. If we do if we use something like WhatsApp is usually a little bit difficult (...)”,Focus group participant 7*

*5.3. Poor integration and interoperability*

*“Each doctor has gone to a different provider and procured something. So, there’s still no integration. There’s not integration with the pharmacy. There’s no integration with the labs. So, I still have to take the prescription myself and go to the pharmacy, or I still have to collect the lab report and bring it back to my doctor, even though that’s all in the same hospital. So, when you think about that across an entire hospital, chain of hospitals, or under our health system, there’s still massive opportunities but also massive gaps”, Interview participant 3*

*“A number of different people I worked for, or I was talking to, were all trying to create their own electronic health record. It’s depressing because it’s so duplicative and you think, ‘why is everybody trying to do this?’ But the reason (…) [is because] there’s not a standard integrated one that could work across different hospitals, across different systems”, Interview participant 3*

***Thematic analysis of opportunities identified***

*1. Patients*

*1.1. Improving literacy*

*“If there was a lot more education about the use of digital healthcare or even just digital generally (…), I think that would be beneficial”, Interview participant 9*

*1.2. Improve patient empowerment and self-care*

*“There are different foundations working on getting some digital tools in the hands of patients. You could imagine small tools that patients can use to measure oxygen levels, blood pressure, and send those metrics to doctors or nurses”, Interview participant 7*

*“We have even spoken to somebody who has a digital stethoscope, so then you think about in the middle of somewhere you have a virtual clinic with a digital stethoscope, and somebody hold it there, and in Nairobi the doctor can hear their heartbeat”, Interview participant 3*

*1.3. Tailored solutions and user-centred design*

*“Some community healthcare workers do not have any level of education. And sometimes it's a bit hard for them to read and write. And when you give them a digital application, training becomes a bit hard. So, one of the things that it's recommended from government is to make these tools as simple as possible to use, incorporate more graphics, such that they are designed with the end user in mind”, Interview participant 12*

*1.4. Improve equity*

*“I believe it has those benefits of bringing more equity and access, and improving health outcomes, it’s only if we’re able to improve the access to truly everyone, right?”, Interview participant 1*

*2. Providers*

*2.1. Enhancing access to secondary care advice*

*“It will also (…) improve quality because you can be able to get a second opinion from specialist doctors using digital health, to be able to support the care in patients so that the quality is almost the same in urban and rural areas", Focus group participant 17*

*"And if we are lucky that the country has a good connection, you can imagine a doctor (...) could be remotely consulted. (...) We could hopefully even have vídeo, so that we can actually see things, rather than just having the voice", Interview participant 7*

*2.2. Clinical support tools*

*"I think that [artificial intelligence] is going to support them [clinicians] in the decision-making process, as they're deciding which tests they want to prescribe to the patient, depending on their symptoms”, Interview participant 6*

*"Artificial intelligence could help even someone who has not been trained recently (…). They could have a tool that enables them to have someone literally telling them, did you check this, this is the latest thing”, Interview participant 7*

*“[We] will be the ability to standardise approaches to care, (…) empower clinicians with a little bit less experience through digital tools, empower them to give more to their patients", Interview participant 6*

*3. Health systems*

*3.1. Improve quality of care*

*“(…) All elements of quality of care can be positively impacted when we use digital health appropriately. (…) If we look at it from the staff perspective, we can use digital health to improve their capacity and their skills and knowledge so that keeps them well motivated. And from the client perspective, we can use it to improve patient satisfaction from many angles: accessibility, financial transparency. So, all this can have a very positive impact on the quality of care received by the clients”, Focus group participant 22*

*"By having these apps, we first digitise and then we utilise all the information for improved patient outcomes and better efficiency", Interview participant 4*

*3.2. Knowledge generation (research, planning and delivery)*

*"Data analytics, and of course leveraging artificial intelligence, can create tailor-made preventative solutions, without the intervention of a human”, Interview participant 8*

*"With data analytics you will be able to come up with new treatment models, even preventative treatment models”, Interview participant 8*

*“With digital health we can actually improve all our data for planning purposes (…) We can get accurate data, and evaluate health system as much as possible”, Interview participant 2*

*3.3. Optimization of resources and finances*

*“(…) for me, one point is that [DHTs] have the potential to shift, especially in Africa context, (…) healthcare or the health systems, from the perspective of an aid corridor into a source of economic empowerment”, Focus group participant 15*

*3.4. Learning from best practice and scaling up*

*“We’re trying to set up systems, pathways, and frameworks for the context [in which] these systems are going to be used. [But] there is no point reinventing the wheel really, if there are things that are working somewhere else, and that can be transposed or adapted. Perhaps those things would help us to take an edge, rather than having to do all the design work from scratch”, Focus group participant 8*

*“There are probably opportunities to look at (…) what works in similar contexts. (…) Even in places like the US, if there are systems and frameworks that can help to design and develop technology, that will be cheap, easily accessible, usable in the context of African health care – why not [use them]?”, Focus group participant 8*

*“They are also building as they go, data privacy exists, data sharing exists, they are building the blocks there. It’s not perfect, of course we need to continue to get more refined (...) And that requires maybe sharing experience of advanced countries that have already done this, so people are not inventing the wheel, but also mindful about the local environment”, Interview participant 7*

*4. Legislation and regulation*

*4.1. Improve governmental support*

*“It would be useful to have more policy engagement (…), especially at the ministry level to kind of guide them through that process.” Interview participant 1*

*4.2. Legal implications*

*“For example, in telemedicine [...] When do you refer the patient? When do you prescribe on [the] phone? What if anything goes wrong? You will be liable as a clinician. So, government will need to regulate such practice, so that we know where to draw the line”, Interview participant 11*

*4.3. Data ownership and monetization*

*“You can trade the underlying value and still get the money for that health information without having to sell the health information. That’s something that we’re looking at for areas where there’s regulatory issues and it’s not really clear.” Interview participant 4*

*5. Technology and infrastructure*

*5.1. Improving basic structure*

*“If [the] government makes power supply stable, digital health will increase geometrically." Interview participant 11*

*5.2. Data linkage*

*“You want to make sure that if you are trying to track the numbers (...) in the country so that you are able to feed back into a national system. So, the linkages between the existing national and brick and mortar facilities and the virtual one, should be linked so that they can exchange the information literally live", Interview participant 7*

*“A unique identifier in your civil registration number identifier should make its way into every piece, from pathology specimen request to every clinical record”, Interview participant 10*

***Thematic analysis of threats identified***

*1. Patients*

*1.1. Patients buy-in*

*“I think that it will take a lot of education and motivation (…) to get people to actually rally behind this whole eHealth agenda that we are seeking to improve primary health care”, Focus group participant 23*

*“So how do we get everyone to buy into it? How do we build a bigger pool of evidence that would mean when we're talking about the usefulness of digital technology? We're talking about strong local evidence, relevant evidence. So perhaps more research into it (…), perhaps publications, perhaps talking not only within the healthcare system [but] lobbying beyond healthcare”, Focus group participant 9*

*2. Providers*

*2.1. Providers buy-in*

*There is a lot of push back from existing doctors”, Interview participant 3*

*2.2. Lack of human resources and local capacity*

*“We collaborate with people outside the country most of the time. I feel the challenge is there’s no local capacity”, Interview participant 2*

*“Nurse and doctor availability has come to [a] plateau. If you look at the trajectory and the future, if you do any projections, particularly on human resourcing in the developing world, if anything the gap will widen and widen over time. The country will be unable to produce enough nurses and doctors to take care of the condition of the population”, Interview participant 7*

*3. Health care systems*

*3.1. Resistance to change*

*“Acceptance of the importance of digital technology for health is probably one of the main threats because it you know; it is fairly new, and it means change. And we all know how difficult it can be to change, especially if we're thinking about a systemic change”, Focus group participant 9*

*“Doctors traditionally are very resistant to change, especially to technology. I'm a physician, I know that many, many of my colleagues don't like computers (...) because they think it comes between them and the patient”, Focus group participant 22*

*3.2. Lack of governmental support*

*“One of the barriers would be political will. A lot of people talk, and they don’t walk their talk - this is amazing, but they don’t truly prioritise it”, Interview participant 9*

*3.3. Fraud and misuse*

*“There’s the whole, how do I know that this is actually the patient? Let’s say, in terms of insurance, someone can just take a phone and pretend to be, so there must be a way to verify the caller against the insurance or whatever card they’re using, for the identification of the patient.”, Interview participant7*

*3.4. Sustainability*

*“We have funding to implement electronic medical records (EMR) for five years, and then the project’s over. We don’t know if they’re actually still using it, or what their [situation is] like. You’re at risk of all that investment and those five years of trying to create an EMR, just to kind of going down the drain”, Interview participant 1*

*“The sustainability of projects – I’ll say digital interventions – is often conformed to the project life cycle of a donor”, Interview participant 1*

*4. Regulations*

*4.1. Restrictive regulations*

*“We are heading a certain way and it could be very easy for all of that to stop with really regressive regulation, and I think that’s by far the biggest threat”, Interview participant 3*

*“And at some point, the regulation will be created and it will probably be far more constricted, that’s what we’re expecting at least, than what systems have been doing, and we will all have to pull back to meet the regulations, probably to the disadvantage of the patient at the end of it”, Interview participant 3*

*5. Infrastructure and technology*

*5.1. Cybersecurity and data privacy*

*“For example, when something happens or when a disease breaks out in a certain area, and we are doing surveillance or something, communities mostly ask you: “Where are you taking our stories? Where are you taking our information? There's a bit of a concern.”, Focus group participant 16*

*“The issue of their medical records being with a third-party, that’s usually a concern”, Interview participant 8*

*“There is this matter of data security, and that would probably be a big issue as well. How do we make sure confidential information remains confidential within the new systems that we put in place? (…) Are the SMS being used in (…) the usual phones? Is it the same network that everyone uses, or is it a secure one? And obviously, it's easier to use the same network that everybody uses. you can already start to think where is the data going to be stored (…), if you want to digitalize the patient's record?”, Focus group participant 8*
